# Supplementary material for: Sustained-input switches for transcription factors and microRNAs are central building blocks of eukaryotic gene circuits
Source: Genome Biol. 2013 Aug 23;14(8):R85. doi: 10.1186/gb-2013-14-8-r85 (PMC4054853; doi:10.1186/gb-2013-14-8-r85)
Supplement: Additional file 5 — HTML Browsable Motif Output. Zipped folder containing all WaRSwap and FANMOD motif output, viewable in a web browser. [file gb-2013-14-8-r85-S5.ZIP › HTML_browsable_motif_output/WaRSwap_ath_tair9/sigs_WSWRSLDraw_2-2000.pvals.heatmaps.html/motif_id_36_001001002_tftype_ath_upstream_-1000_0.html]

```
BG_MODEL = Rnew2
MOTIF_ID = 36_001001002
TF_TYPE = ath
UPSTREAM = -1000_0


PVals
FN_0.2	FN_0.4	FN_0.6	FN_0.8
dg_60.genes	0.594	0.985	0.985	0.008
dg_70.genes	0.601	0.993	0.984	0.009
dg_80.genes	0.593	0.987	0.98	0.004

ZScores
FN_0.2	FN_0.4	FN_0.6	FN_0.8
dg_60.genes	-0.296	-2.06	-2.047	2.018
dg_70.genes	-0.311	-2.169	-1.996	1.964
dg_80.genes	-0.287	-2.079	-2.072	2.026

StDevs
FN_0.2	FN_0.4	FN_0.6	FN_0.8
dg_60.genes	28880.666	35870.334	8869.273	1522.078
dg_70.genes	28836.145	33656.92	8843.423	1585.457
dg_80.genes	28731.662	34330.778	8587.87	1552.624
```
